# Factors affecting perforation of the esophagus in patients with deep neck infection

**DOI:** 10.1186/s12879-022-07480-6

**Published:** 2022-05-27

**Authors:** Shih-Lung Chen, Chia-Ying Ho, Shy-Chyi Chin, Yu-Chien Wang

**Affiliations:** 1 Department of Otorhinolaryngology & Head and Neck Surgery, Chang Gung Memorial Hospital, Linkou, Taiwan; 2 School of Medicine, Chang Gung University, Taoyuan, Taiwan; 3 Division of Chinese Internal Medicine, Center for Traditional Chinese Medicine, Chang Gung Memorial Hospital, Taoyuan, Taiwan; 4 Department of Medical Imaging and Intervention, Chang Gung Memorial Hospital, Linkou, Taiwan; 5 Department of Otorhinolaryngology & Head and Neck Surgery, New Taipei Municipal TuCheng Hospital (Built and Operated By Chang Gung Medical Foundation), New Taipei, Taiwan

**Keywords:** Deep neck infection, Esophageal perforation, Mediastinitis, Retropharyngeal space

## Abstract

**Background:**

Deep neck infection (DNI) is a serious disease that can lead to severe morbidity, including esophageal perforation, and mortality. However, no previous study has explored the risk factors associated with esophageal perforation in patients with DNI. This study investigated these factors.

**Methods:**

Between September 2015 and September 2021, 521 patients with DNI were studied. Relevant clinical variables and deep neck spaces were assessed.

**Results:**

In a multivariate analysis, involvement of the retropharyngeal space (OR 5.449, 95% CI 1.603–18.51, *p* = 0.006) and the presence of mediastinitis (OR 218.8, 95% CI 55.98–855.3, *p* < 0.001) were independent risk factors associated with esophageal perforation in patients with DNI.

There were no differences in pathogens between 32 patients with and 489 patients without esophageal perforation (all *p* > 0.05).

**Conclusion:**

Involvement of the retropharyngeal space and the presence of mediastinitis were independent risk factors associated with esophageal perforation in patients with DNI. There were no differences in pathogens between the groups with and without esophageal perforation in DNI.

## Introduction

Deep neck infection (DNI) is a severe bacterial infection in the potential spaces of the neck [1]. DNI can lead to airway obstruction and causes severe morbidity, including esophageal perforation, sepsis, descending necrotizing mediastinitis, necrotizing fasciitis, disseminated intravascular coagulation, carotid artery erosion, jugular vein thrombosis, pericarditis, and pleural empyema [2–8]. The mortality rate is 40–50% if such complications occur [9].

Esophageal perforation is a potentially life-threatening condition. Timely surgical incision and drainage of an abscess, esophageal repair, adequate intravenous antibiotic therapy, and enteral or parenteral nutrition are critical for an improved prognosis [10, 11]. Previous research has studied DNI from esophageal perforation caused by a foreign body [12–14].

However, no studies have explored the risk factors associated with esophageal perforation in patients with DNI. This research investigated these risk factors.

## Materials and methods

This study retrospectively reviewed the medical records of 521 patients diagnosed with DNI who were admitted to Chang Gung Memorial Hospital in Linkou, Taiwan, between September 2015 and September 2021. Diagnostic imaging procedures included ultrasonography (US) and computed tomography (CT). Treatment included antibiotics, US-guided needle drainage, and open surgical incision and drainage. The empirical antibiotics used were ceftriaxone (1 g q12h) and metronidazole (500 mg q8h), according to previous reports, to cover aerobic and anaerobic bacteria before the culture results were available [15, 16]. Enteral or parenteral nutrition feeding was given if esophageal perforation was suspected. All esophageal perforations were confirmed by a swallow study (esophagogram) with a barium swallow [17].

For the patient with small esophageal perforation and limited extra-esophageal involvement, we used conservative management including oxygen supplement, large bore intravenous access and cardiopulmonary monitoring. We kept the patient nothing per oral and a nasogastric tube placed for feeding and limit possible contamination. Broad spectrum intravenous antibiotics were be given with adequate analgesia [18].

Although there is no definite recommendation for indication of surgery [19], we consulted thoracic surgeon for surgical evaluation when esophageal perforation occurred with hemodynamic instability or serious extravasations of contrast into adjacent body cavities.

Basically, we judged whether esophageal perforation caused mediastinitis or mediastinitis resulted in esophageal perforation based on the time of medical history, the flow of esophagogram contrast and CT presentation.

To investigate the risk factors associated with an esophageal perforation, we collected the following patient data: gender, age, chief complaint period, hospital-staying period, C-reactive protein (CRP) level, blood sugar, diabetes mellitus (DM) status, performance of incision and drainage surgery, results of US-guided drainage, number of spaces affected by DNI, involvement of deep neck spaces, presence of mediastinitis, and esophageal perforation.

### Exclusion criteria

Patients with a history of swallowing a foreign body, severe cardiopulmonary disease, immunocompromised condition, previous head and neck tumor surgery, or previous esophageal tumor surgery were excluded. A total of 521 patients were included.

### Statistical analysis

All data were analyzed using MedCalc software (ver. 18.6; Ostend, Belgium). The Kolmogorov–Smirnov test showed that the data were not normally distributed; thus, we employed the chi-square test for categorical variables, the Mann–Whitney U test to compare continuous variables, and logistic regression for the univariate and multivariate analyses. A multivariate forward stepwise selection procedure was implemented, and all variables included in the univariate analysis were entered into the final multivariate model. A *p* value < 0.05 was considered to reflect statistical significance.

## Results

Demographic and clinical data are shown in Table 1. A total of 521 patients with DNI were included; 340 males (65.25%) and 181 females (34.75%), with a mean age of 51.82 ± 19.21 years. The mean chief complaint and hospitalization periods were 4.84 ± 4.23, and 10.02 ± 8.33 days, respectively. For the laboratory data, the mean CRP level was 147.63 ± 107.07 mg/L, and the mean blood sugar level was 145.10 ± 73.46 mg/dL. A total of 214 (41.07%) patients had DM.

**Table 1:** Clinicopathological characteristics of the 521 patients with deep neck infection

Characteristics	N (%)
Gender	521 (100.00)
Male	340 (65.25)
Female	181 (34.75)
Age, years (SD)	51.82 ±19.21
Chief complaint period, days (SD)	4.84 ± 4.23
Hospital-staying period, days (SD)	10.02 ± 8.33
CRP, mg/L (SD)	147.63 ± 107.07
Blood sugar, mg/dL (SD)	145.10 ± 73.46
Diabetes mellitus	214 (41.07)
Incision & drainage open surgery	249 (47.79)
Ultrasonography-guided drainage	86 (16.50)
Single space	188 (36.08)
Double spaces	161 (30.90)
Multiple spaces, ≥ 3	172 (33.01)
Deep neck space involvement	
Retropharyngeal space	174 (33.39)
Parapharyngeal space	310 (59.50)
Submandibular space	251 (48.17)
Masticator space	125 (23.99)
Anterior cervical space	41 (7.86)
Parotid space	88 (16.89)
Perivertebral space	20 (3.83)
Carotid space	36 (6.90)
Visceral space	34 (6.52)
Posterior cervical space	10 (1.91)
Mediastinitis	45 (8.63)
Esophageal perforation	32 (6.14)

*N* numbers; *SD* standard deviation; *CRP* C-reactive protein (normal range < 5 mg/L); Sugar (normal range: 70–100 mg/dL)

For DNI treatment procedures, 249 patients (47.79%) underwent incision and drainage, and 86 (16.50%) underwent an US-guided drainage procedure.

Among these patients, 188 (36.08%) had single space involvement, 161 (30.90%) had double space involvement, and 172 (33.01%) had ≥ 3 spaces involved.

Regarding deep neck space involvement, 174 (33.39%) patients had retropharyngeal spaces involved, 310 (59.50%) had parapharyngeal spaces involved, 251 (48.17%) had submandibular spaces involved, 125 (23.99%) had masticator spaces involved, 41 (7.86%) had anterior cervical spaces involved, 88 (16.89%) had parotid spaces involved, 20 (3.83%) had perivertebral spaces involved, 36 (6.90%) had carotid spaces involved, 34 (6.52%) had visceral spaces involved, and 10 (1.91%) patients had posterior cervical spaces involved. Mediastinitis was found in 45 (8.63%) patients. Esophageal perforation was found in 32 (6.14%) patients.

In Table 2, we performed univariate analysis of variables for 521 patients with DNIs and found that a higher CRP level, multiple spaces (≥ 3 spaces), involvement of the retropharyngeal space, and the presence of mediastinitis were significantly associated with esophageal perforation (*p* < 0.05).

**Table 2:** Univariate and multivariate analysis of esophageal perforation in 521 patients with deep neck infection

Variable	Esophageal perforation		Univariate analysis			Multivariate analysis		
	Yes	No	OR	95% CI	*p* value	OR	95% CI	*p* value
Gender	32	489			0.272	–	–	–
Male	18	322	1.000					
Female	14	167	1.499	0.727–3.090				
Age, years					0.413	–	–	–
≤ 50	13	235	1.000					
> 50	19	254	1.353	0.653–2.801				
Chief complaint days (SD)	3.81 ± 2.44	4.90 ± 4.31	0.909	0.796–1.037	0.102	–	–	–
CRP, mg/L (SD)	187.56 ± 107.89	145.01 ± 106.61	1.003	1.000–1.006	0.031*	–	–	–
Blood sugar, mg/dL (SD)	156.15 ± 88.16	144.38 ± 72.44	1.001	0.997–1.006	0.401	–	–	–
Diabetes mellitus					0.156	–	–	–
No	15	292	1.000					
Yes	17	197	1.679	0.819–3.442				
Multiple spaces, ≥ 3					< 0.001*	–	–	–
No	12	337	1.000					
Yes	20	152	3.695	1.761–7.751				
Retropharyngeal space					< 0.001*			0.006*
No	6	341	1.000					
Yes	26	148	9.984	4.025–24.76		5.449	1.603–18.51	
Parapharyngeal space					0.262	–	–	–
No	16	195	1.000					
Yes	16	294	1.507	0.736–3.085				
Submandibular space					0.103	–	–	–
No	21	249	1.000					
Yes	11	240	1.841	0.868–3.898				
Masticator space					0.890	–	–	–
No	24	372	1.000					
Yes	8	117	1.059	0.463–2.422				
Anterior cervical space					0.349	–	–	–
No	28	452	1.000					
Yes	4	37	1.745	0.580–5.242				
Parotid space					0.774	–	–	–
No	26	407	1.000					
Yes	6	82	1.145	0.457–2.870				
Perivertebral space					0.823	–	–	–
No	31	470	1.000					
Yes	1	19	1.253	0.162–9.671				
Carotid space					0.078	–	–	–
No	27	458	1.000					
Yes	5	31	2.736	0.985–7.596				
Visceral space					0.202	–	–	–
No	28	459	1.000					
Yes	4	30	2.185	0.719–6.638				
Posterior cervical space					0.635	–	–	–
No	31	480	1.000					
Yes	1	9	1.720	0.211–14.07				
Mediastinitis					< 0.001*			< 0.001*
No	5	471	1.000					
Yes	27	18	141.3	48.75–409.4		218.8	55.98–855.3	

*SD* standard deviation; *OR* odds ratio; *CI* confidence intervals; *CRP* C-reactive protein

*p < 0.05. Significant differences are shown in bold

Patients with esophageal perforation had an average mean CRP level of 187.56 ± 107.89 mg/L. The CRP level for those without esophageal perforation was 145.01 ± 106.61 mg/L (OR 1.003, 95% CI 1.000–1.006, *p* = 0.031). Multiple space involvement (≥ 3 spaces) was a significant risk factor for esophageal perforation (OR 3.696, 95% CI 1.761–7.751, *p* < 0.001). Involvement of the retropharyngeal space was also a risk factor for esophageal perforation (OR 9.984, 95% CI 4.025–24.76, *p* < 0.001). The presence of mediastinitis was another significant risk factor for esophageal perforation (OR 141.3, 95% CI 48.75–409.4, *p* < 0.001).

In Table 2, all factors were entered into a forward stepwise multivariate logistic regression model. Involvement of the retropharyngeal space (OR 5.449, 95% CI 1.603–18.51, *p* = 0.006) and the presence of mediastinitis (OR 218.8, 95% CI 55.98–855.3, *p* < 0.001) were significant independent risk factors for esophageal perforation in patients with DNI.

Table 3 shows a comparison of pathogens between 32 patients with and 489 patients without esophageal perforation. There were no significant differences in pathogens between these two groups (all *p* > 0.05). In the esophageal perforation group, only two patients (6.25%) had no growth of specific pathogens.

**Table 3:** Comparison of pathogens between 32 patients with esophageal perforation and 489 patients without esophageal perforation in deep neck infection

Pathogens	Perforation, N (%)	Non-perforation, N (%)	*p* value
*Streptococcus constellatus*	10 (31.25)	107 (21.88)	0.272
*Streptococcus anginosus*	8 (25.00)	80 (16.35)	0.222
*Klebsiella pneumonia*	8 (25.00)	61 (12.47)	0.056
*Prevotella buccae*	6 (18.75)	49 (10.02)	0.133
*Prevotella intermedia*	5 (15.62)	40 (8.17)	0.182
*Staphylococcus aureus*	4 (12.50)	21 (4.29)	0.059
*Parvimonas micra*	3 (9.37)	39 (7.97)	0.735
*Staphylococcus epidemidis*	2 (6.25)	10 (2.04)	0.164
*Pseudomonas aeruginosa*	2 (6.25)	9 (1.84)	0.142
*Eikenella corrodens*	2 (6.25)	8 (1.63)	0.120
*Streptococcus salivarius*	1 (3.12)	10 (2.04)	0.505
*Streptococcus oralis*	1 (3.12)	8 (1.63)	0.437
*Slackia exigua*	0 (0.00)	14 (2.86)	1.000
*Gemella morbillorum*	0 (0.00)	13 (2.65)	1.000
No growth	2 (6.25)	101 (20.65)	0.063

*N* number

## Discussion

DNI often occurs following preceding infections such as a peritonsillar abscess, pharyngitis, or odontogenic infection [20, 21]. Clinical management usually involves departmental coordination between the general ward, intensive care unit, and operating room, with a multidisciplinary approach consisting of the otolaryngologist, chest surgeon, and anesthetist [22]. Our research found that involvement of the retropharyngeal space and the presence of mediastinitis were independent risk factors associated with esophageal perforation in patients with DNI. There were no differences in pathogens between the groups with and without esophageal perforation.

In Table 1, the male predominance was also observed in previous studies [15, 23]. The average age of our patients was similar to previous studies [16].

In Table 2, compared with those who did not have esophageal perforation, the patients with esophageal perforation had a higher average mean CRP level, which achieved statistical significance on univariate analysis (*p* < 0.031).

CRP is an acute inflammatory protein released during infectious processes. Wang et al*.* reported that patients with DNI and a CRP level > 100 mg/L have longer hospital stays [24].

However, CRP did not reach statistical significance in our multivariate analysis. Our univariate analysis results indicated that DNI involving multiple spaces (≥ 3 spaces) is a risk factor for esophageal perforation. In one study, the infection was lethal when DNI involved multiple spaces [1]. Furthermore, involvement of multiple deep neck spaces was a risk factor for patients to undergo tracheostomy [9]. However, multiple spaces (≥ 3 spaces) was not an independent risk factor in our multivariate analysis.

We considered higher levels of CRP and involvement of multiple spaces were representative of more severe infection, but they did not necessarily mean that DNI would lead to esophageal involvement or esophageal perforation. If a severe abscess did not occur at a critical site and invade the esophagus, esophageal perforation almost never happened, even with a high CRP level or involvement of multiple spaces.

**Fig. 1:**
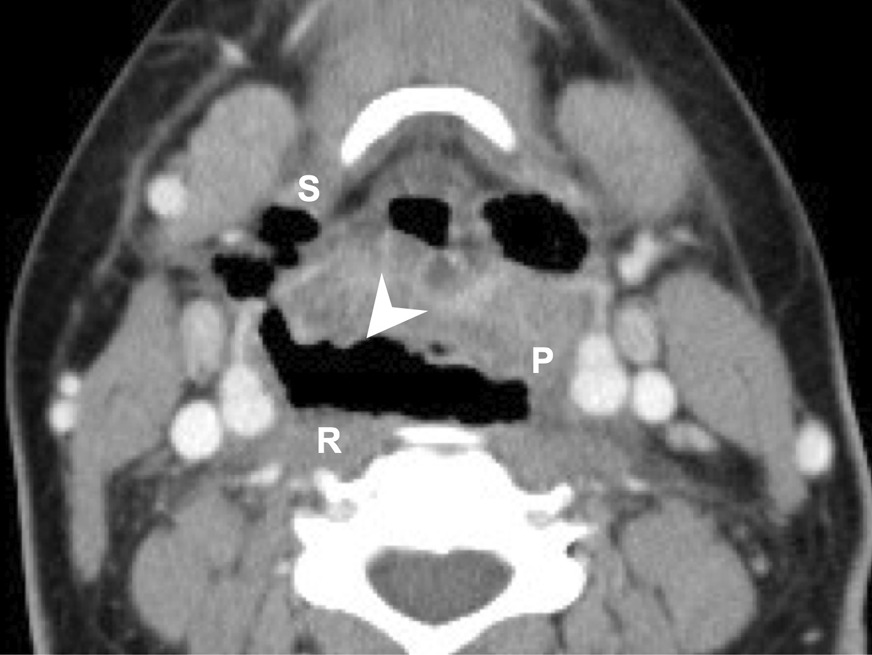
The axial CT view of a patient with a deep neck infection and esophageal perforation. *R* retropharyngeal space; *P* parapharyngeal space; *S* submandibular space; air dissection (arrowhead)

The deep neck spaces lie within a complex framework formed by the cervical fascial planes [25]. The retropharyngeal space is a potential space of the head and neck just behind the esophagus, bound by the buccopharyngeal fascia anteriorly and the alar fascia posteriorly. Serious infections of the teeth can spread down this space into the posterior mediastinum [26]. Our research found that the retropharyngeal space is an independent risk factor for esophageal perforation in DNI. With retropharyngeal space involvement, the esophagus may become distorted and compressed, which can further lead to perforation (Fig. 1).

**Fig. 2:**
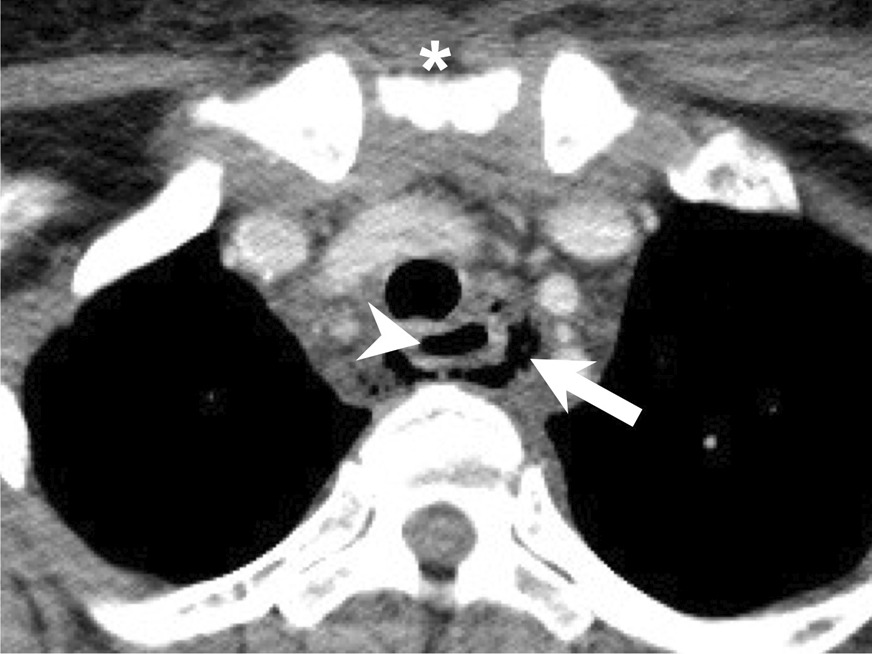
Severe mediastinitis in a patient with esophageal perforation. Esophagus (arrowhead); air dissection (arrow); sternal notch (asterisk)

In our study, 8.63% of patients developed mediastinitis (Table 1). The presence of mediastinitis was a significant risk factor in both univariate and multivariate analyses for esophageal perforation (Table 2). Previous reports also showed that esophageal perforation and mediastinitis usually occurred together [27, 28]. When the mediastinum is severely infected with micro-abscesses and gas formation (Fig. 2), the esophagus is invaded and perforation or rupture becomes possible. The mortality rate of an infective mediastinal extension can reach around 40% [29]. From our research, the location (retropharyngeal space and mediastinum) is the most significant element for esophageal perforation in DNI. It confirms that the key infection site is highly associated with relevant complication.

**Fig. 3:**
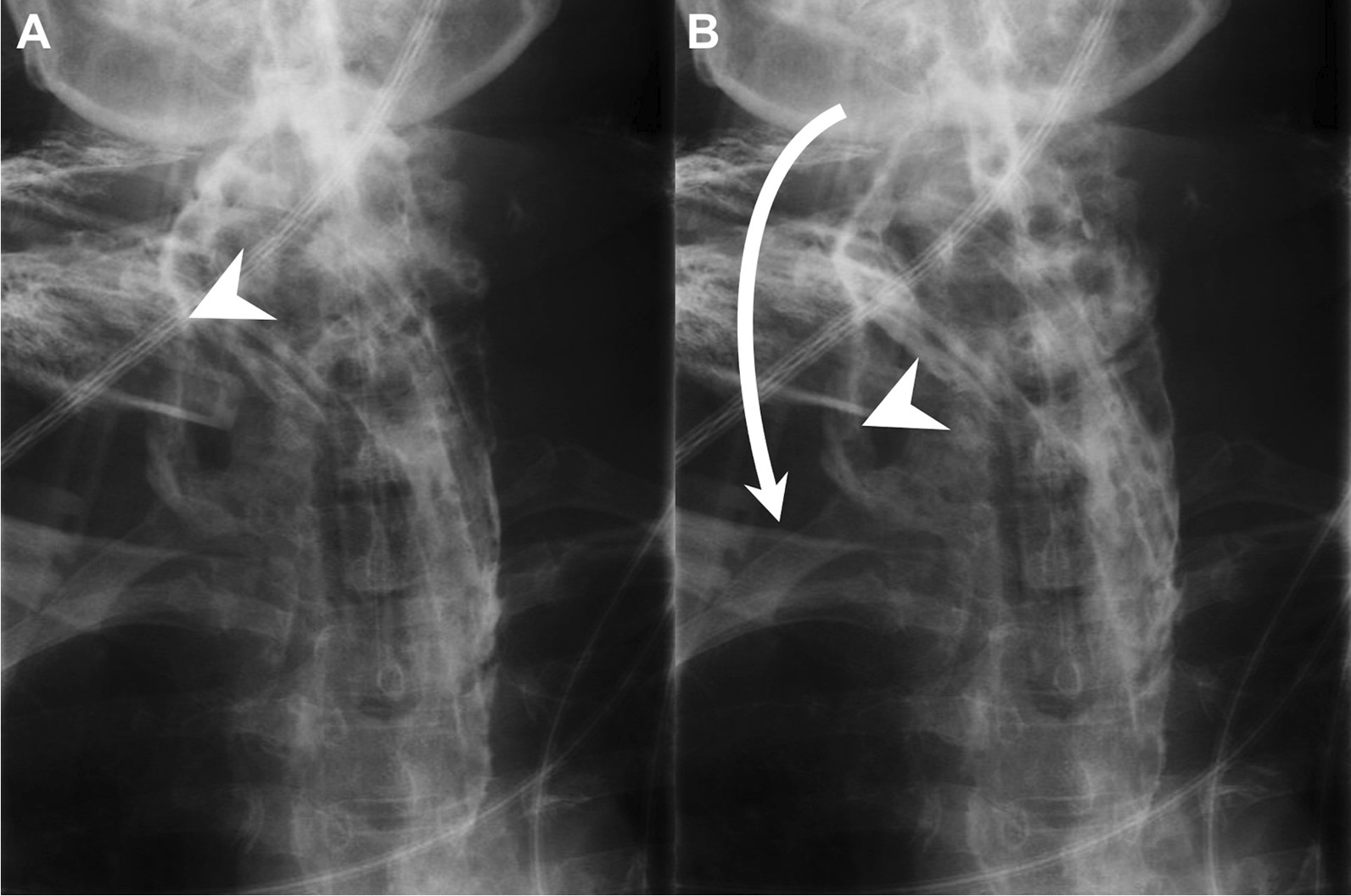
**A–B** Contrast leakage from the site of esophageal perforation in a patient with deep neck infection on a swallow study (esophagogram). Contrast (arrowhead); flow direction (arrow line)

Prompt clinical suspicion and appropriate imaging are important for the management of esophageal perforation. In this cohort, the esophageal perforation rate was 6.14%. A swallow study provides a radiological evaluation of the esophagus and can diagnose structural diseases and motility disorders of the esophagus [30]. The swallow study remains the gold standard study for esophageal perforation [31], and the leakage of contrast can confirm the diagnosis (Fig. 3) [11]. In addition to a swallow study, esophageal perforation can be evaluated by a CT scan [32]. Surgical repair remains an important option for many patients, but a non-operative approach, with or without the use of an endoscopic stent should be considered when the clinical situation allows for a less invasive procedure [11]. The primary treatment for esophageal perforation with DNI includes nothing by mouth, effective broad-spectrum antibiotics against the causative organisms, and enteral or parenteral nutrition. The most common cause of mortality due to esophageal perforation is multiorgan failure resulting from sepsis [33]. A repeat swallow study is necessary. If the exam shows resolution or improvement of the perforation, oral intake can be initiated [32].

In Table 3, there was no statistical difference in pathogens between 32 patients with and 489 patients without esophageal perforation in DNI. DNIs have various clinical presentations depending on the pathogenic organism [34]. *Streptococcus constellatus* was the most frequently cultivated pathogen in patients, regardless of whether there was an esophageal perforation (31.25%) or not (21.88%). This organism, which belongs to the *Streptococcus milleri* group, is commonly found on the mucous membranes of the oral cavity and oropharynx. Although it behaves as a commensal organism, it can become invasive and pathogenic after mucosal disruption, cause infection and abscesses, and lead to a locally aggressive extension to surrounding tissues such as the deep neck spaces [34–36].

### Limitations of the article

There were some limitations to our study. The retrospective design of this study gave rise to a certain attrition rate. The majority of the study population was male, which could be a selection bias, but this is common in retrospective studies.

## Conclusion

Involvement of the retropharyngeal space and the presence of mediastinitis were independent risk factors associated with esophageal perforation in patients with DNI. There were no differences in pathogens between the groups with and without esophageal perforation in DNI.

## Data Availability

All data generated or analyzed during this study are included in this published article. The data are available on request. The English in this document has been checked by at least two professional editors, both native speakers of English. For a certificate, please see: http://www.textcheck.com/certificate/4HGgVV.

## Abbreviations

DNI: Deep neck infection
US: Ultrasonography
CT: Computed tomography
CRP: C-reactive protein
DM: Diabetes mellitus
OR: Odds ratio
